# The Association Between Gut Microbiome and Cachexia in Colorectal Cancer: A Systematic Review

**DOI:** 10.3390/cancers18152387

**Published:** 2026-07-24

**Authors:** Mariem Hachani, Tafirenyika Gwenzi, Ben Schöttker, Jens Puschhof, Christoph Stein-Thöringer, Gianni Panagiotou, Hermann Brenner, Michael Hoffmeister

**Affiliations:** 1 Division of Clinical Epidemiology of Early Cancer Detection, German Cancer Research Center (DKFZ), 69120 Heidelberg, Germany; mariem.hachani@dkfz-heidelberg.de (M.H.); tafirenyika.gwenzi@dkfz-heidelberg.de (T.G.); b.schoettker@dkfz-heidelberg.de (B.S.); 2 Medical Faculty Heidelberg, Heidelberg University, 69120 Heidelberg, Germany; 3 Junior Research Group Epithelium Microbiome Interactions, German Cancer Research Center (DKFZ), 69120 Heidelberg, Germany; jens.puschhof@dkfz-heidelberg.de; 4 Internal Medicine I, Translational Microbiome Research Group, University Hospital Tübingen, 72076 Tübingen, Germany; christoph.stein-thoeringer@uni-tuebingen.de; 5 Microbiome Dynamics, Leibniz Institute for Natural Product Research and Infection Biology—Hans Knöll Institute, 07745 Jena, Germany; gianni.panagiotou@leibniz-hki.de; 6 Cluster of Excellence Balance of the Microverse, Friedrich Schiller University Jena, 07743 Jena, Germany; 7 Department of Medicine, State Key Laboratory of Pharmaceutical Biotechnology, University of Hong Kong, Hong Kong 999077, China; 8 Faculty of Biological Sciences, Friedrich Schiller University, 07743 Jena, Germany; 9 Jena University Hospital, Friedrich Schiller University, 07747 Jena, Germany; 10 Cancer Prevention Graduate School, German Cancer Research Center (DKFZ), 69120 Heidelberg, Germany; h.brenner@dkfz.de

**Keywords:** colorectal cancer, cachexia, gut microbiota, microbiome, muscle wasting, systemic inflammation, dysbiosis

## Abstract

**Background/Objectives**: Colorectal cancer (CRC) is complicated by cachexia, a wasting syndrome with muscle and fat loss that worsens survival and treatment outcomes. Evidence suggests that the gut microbiome may contribute to CRC cachexia, but its role remains unclear. This systematic review synthesizes evidence to identify microbial signatures linked to cachexia hallmarks in CRC. **Methods**: The protocol was pre-registered with PROSPERO and followed PRISMA guidelines. PubMed, Web of Science, and Scopus were searched for studies on CRC patients and preclinical models with cachexia. Eligible studies compared microbiota composition between cachectic and non-cachectic groups to identify alterations linked to cachexia progression. Study quality was assessed using the Newcastle–Ottawa Scale and CAMARADES checklist. **Results**: Of 2456 records, 15 studies met inclusion criteria, including 13 preclinical studies and 2 clinical studies. Study quality was moderate for preclinical studies and high for clinical cohort studies. Murine CRC cachexia models showed reduced alpha diversity and beta diversity shifts. Butyrate-producing taxa were depleted, whereas Enterobacteriaceae and other pathobionts were enriched. Some microbial changes were independent of food intake and linked to inflammation, metabolic dysregulation, and muscle wasting. Clinical evidence was limited to two reports from the same cohort, in which higher pre-surgical abundance of *Fusobacterium nucleatum* and lower *Porphyromonas* and *Actinomyces* spp. were associated with cachexia onset. **Conclusions**: Current evidence suggests an association between CRC-associated cachexia and gut microbiome alterations, but causality, directionality, and clinical relevance remain uncertain.

## Simple Summary

Cachexia is a serious complication of colorectal cancer that causes unintended weight loss, muscle wasting, inflammation, and poorer outcomes. This systematic review examined whether changes in the gut microbiome may be linked to colorectal cancer-associated cachexia. Most available studies had been conducted in mouse models and showed reduced microbial diversity, loss of potentially beneficial bacteria, and increases in bacteria associated with inflammation. Only two studies in patients were available, both from the same cohort. Overall, the findings suggest that gut microbiome changes may be associated with colorectal cancer-associated cachexia, but it remains unclear whether these changes contribute to cachexia or mainly occur as a consequence of the disease.

## 1. Introduction

Colorectal cancer (CRC) is a common and life-threatening malignancy of the digestive system, representing a major challenge to global public health [1]. CRC is the third most frequently diagnosed cancer and the second-leading cause of cancer deaths worldwide, with over 1.9 million new cases and more than 900,000 deaths in 2022 [2].

CRC is strongly associated with cachexia, a multifactorial syndrome characterized by involuntary weight loss, both skeletal muscle and adipose tissue wasting, systemic inflammation, and metabolic alterations [3,4]. Cachexia affects approximately 50–61% of patients with advanced CRC and is a major contributor to morbidity and mortality in this population, whereas its overall prevalence in early-stage CRC patients is substantially lower [3]. This syndrome is associated with reduced tolerance to anticancer therapies, higher risk of treatment side effects, impaired quality of life, and worse overall survival [5]. A recent meta-analysis of gastrointestinal cancers including CRC found a 50% increased risk of death among patients with cachexia compared to non-cachectic patients [6].

Recent research has begun to uncover a connection between cachexia and the gut microbiota, consisting of trillions of microorganisms including bacteria, archaea, viruses, and fungi, playing a crucial role in digestion, immunity, and metabolism [7]. Several previous studies have found that tumor cachexia is characterized by a reduction in beneficial short-chain fatty acid (SCFA)-producing bacteria (such as those from the Lachnospiraceae and Ruminococcaceae families) and by an overgrowth of pro-inflammatory pathobionts, including certain Enterobacteriaceae [8,9].

Furthermore, diverse associations between gut microbes and CRC have been reported. Dysbiosis, an imbalance in the gut microbial ecosystem, has been consistently associated with an increased CRC risk [10]. Pathogenic bacteria such as *Fusobacterium nucleatum* (*Fn*), enterotoxigenic *Bacteroides fragilis*, and colibactin-producing *Escherichia coli* may contribute to CRC by triggering chronic inflammation, generating genotoxins, and promoting immune evasion. While these associations and experimental findings are compelling, their causal relevance to human CRC initiation and progression has not yet been firmly established [11,12,13].

Beyond specific bacterial taxa, microbial metabolites may provide an important link between gut dysbiosis, inflammation, and host metabolic regulation in CRC [14,15]. SCFAs, including butyrate, acetate, and propionate, are produced by bacterial fermentation of dietary fibers and can influence intestinal barrier integrity, immune regulation, and epithelial metabolism [14,15,16]. A reduction in SCFA-producing bacteria may weaken epithelial barrier function and increase gut permeability, allowing microbial products such as lipopolysaccharide (LPS) to enter the circulation and activate toll-like receptor 4 (TLR4)-mediated inflammatory signaling [15,17]. Inflammatory mediators such as interleukin-6 (IL-6) and tumor necrosis factor-alpha (TNF-α) may then promote systemic inflammation and activate pathways relevant to muscle catabolism, including nuclear factor kappa B (NF-κB) and signal transducer and activator of transcription 3 (STAT3)-related signaling [18,19]. In addition, altered bile acid (BA) metabolism and microbial-derived amino acid metabolites, including tryptophan-related metabolites, may influence metabolic signaling, immune responses, and CRC progression [14,15].

Although the gut microbiome has been increasingly studied in relation to CRC development and progression, specific interactions between gut microbes and cachexia in CRC remain underexplored. Therefore, this systematic review aims to synthesize existing evidence on their associations.

## 2. Materials and Methods

### 2.1. Objectives

The primary objective of this systematic review was to summarize existing studies on the gut microbiota composition and diversity associated with CRC-related cachexia hallmarks: weight loss, inflammation, and metabolism alterations.

### 2.2. Literature Search Strategy

This systematic review was pre-registered with the International Prospective Register of Systematic Reviews (PROSPERO CRD420251048700). A literature search was conducted using PubMed, Web of Science, and Scopus databases to identify studies correlating the gut microbiome with cachexia in CRC. The final database search was conducted in May 2026. Database alerts and recently published records were checked before submission to identify any additional eligible studies. Keywords including (“Colorectal cancer” AND “Gut microbiome”) OR (“Gut microbiota” AND “Cachexia”) were searched. Medical subject headings (MeSH) terms were used for the database searches (Table S1). This systematic review followed the recommendations of the Preferred Reporting Items for Systematic Reviews and Meta-Analyses (PRISMA) [20].

### 2.3. Study Selection Criteria

After removing duplicate records, two researchers (MaH, TG) independently screened the title and abstract of the studies. Articles were full-text reviewed if their title and abstract pointed to original analyses in cachectic vs. non-cachectic groups of either CRC patients or from preclinical studies, including the evaluation of the gut microbiota composition through microbiome analysis approaches (e.g., 16S rRNA gene sequencing, shotgun metagenomics). Studies not included in the systematic review were those lacking a control group, using inappropriate animal models of CRC cachexia, comparing a treatment group to a cachectic group, as well as articles not published in English. The grey literature, including dissertations, theses, and conference abstracts, was not searched because the review focused on peer-reviewed studies with sufficient methodological detail and extractable microbiome data.

### 2.4. Data Extraction and Synthesis of Evidence

The findings from the included studies were synthesized by a narrative and thematic approach, as a meta-analysis was not feasible due to substantial heterogeneity. In particular, the lack of standardization in sequencing regions, the use of OTU- versus ASV-based approaches, different reference databases, variable taxonomic resolution, and diverse alpha- and beta-diversity indices prevented meaningful pooling of data for statistical meta-analysis. Key study characteristics were extracted and summarized in tabular form, including study design, country of origin, population, sample size, cachexia definition or assessment, tumor model, tumor cell line, and microbiota analysis methods. To synthesize the evidence, we compared microbial changes across studies, focusing on results of frequent shifts of gut microbiota compositions and diversity associated with cachexia in CRC patients and mouse models. We compiled a comparative table that summarizes increases and decreases in specific bacterial taxa and integrates taxonomic shifts at various levels (phylum, family, genus, and species) along with quantitative data (odds ratios, q-values, *p*-values). The tables serve as a core component of our narrative synthesis, allowing structured comparison across studies. Data extraction was independently performed by Ma.H. and T.G. Any discrepancies were resolved through discussion with M.H.

### 2.5. Study Quality

For the included pre-clinical studies, quality assessment was conducted by two reviewers independently (Ma.H., T.G.) using the CAMARADES checklist (Collaborative Approach to Meta-Analysis and Review of Animal Data from Experimental Studies), a tool specifically designed for evaluating the methodological quality of preclinical studies with a 10 item checklist [21].

Regarding the clinical studies, we used the Newcastle–Ottawa Scale (NOS) for quality assessment across three domains: selection (maximum 4 stars), comparability (maximum 2 stars), and outcome (maximum 3 stars), for a total possible score of 9 stars. Each item within these domains was awarded a star if the study met predefined criteria, with higher star counts indicating higher quality and lower risk of bias.

## 3. Results

### 3.1. Literature Review Results

**Figure 1:**
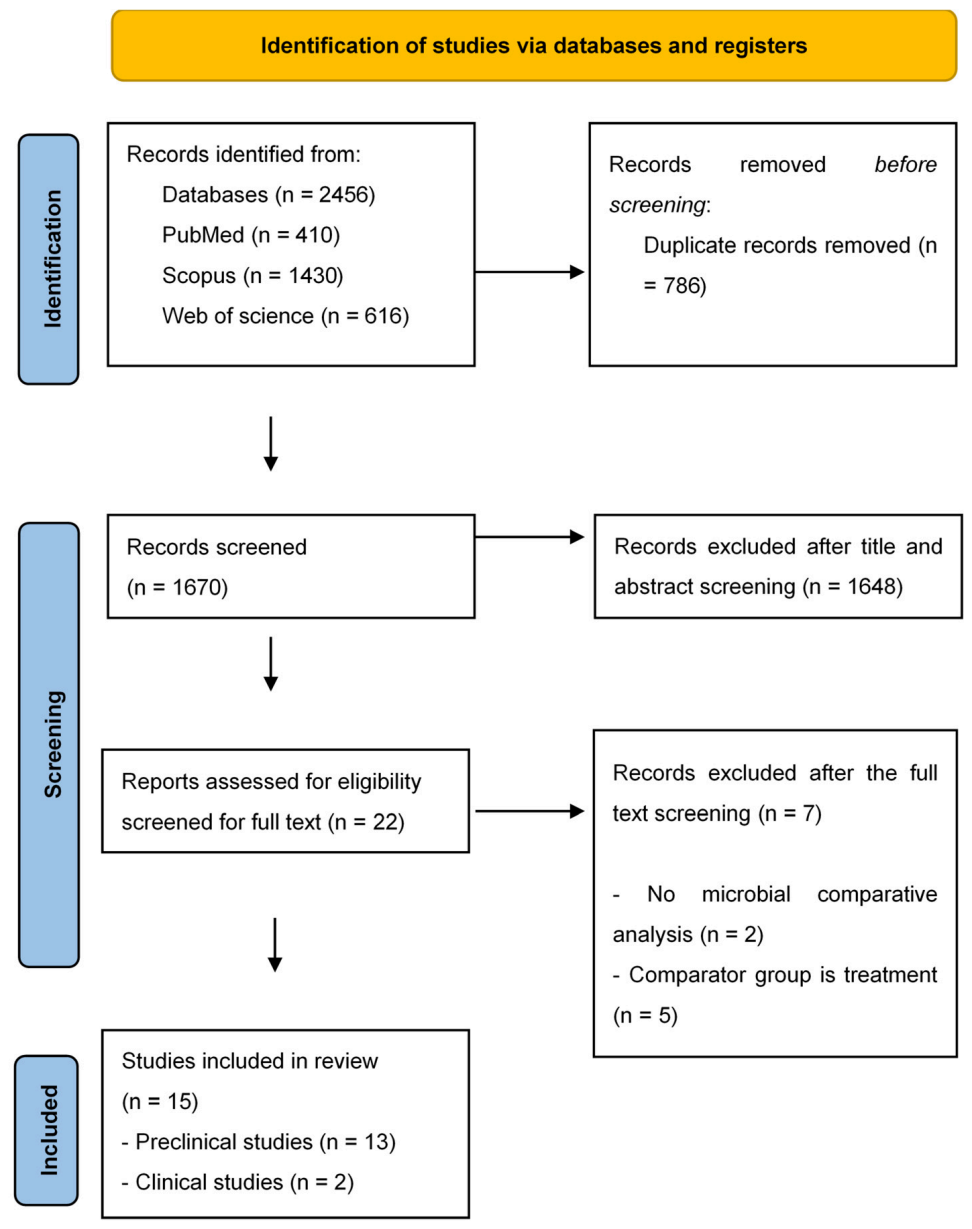
PRISMA Flow Diagram.

Comprehensive database searches identified 2456 records (Figure 1), from which 786 duplicate records were excluded. Of the remaining 1670 records screened by title and abstract, 1648 records were excluded because they did not meet the inclusion criteria due to studies not clearly defining cachexia, or studies unrelated to CRC by using inappropriate mouse models or not including CRC patients. Full texts of all 22 remaining articles were reviewed; after 7 were excluded (Table S2) [22,23,24,25,26,27,28], 15 studies fulfilled the eligibility criteria.

### 3.2. Study Selection and Characteristics

Table 1 presents the primary characteristics of 13 eligible preclinical studies investigating gut microbiome in cancer cachexia murine models. Geographically, the studies were conducted across Europe and Asia. The majority of studies were carried out in Belgium [29,30,31,32,33], the United Kingdom [29,32,33], France [29,30], and Finland [34]. In Asia, studies were conducted in China [35,36,37,38,39,40] and Korea [41]. The two clinical studies were conducted in the USA and Germany, respectively [42,43] (Table 2). Across the preclinical studies, gut microbiota composition was predominantly assessed using 16S rRNA gene sequencing [29,34,35,38,41], with several studies also employing quantitative PCR (qPCR) to validate specific bacterial taxa [30,31]. Some studies combined sequencing with metabolomics, including SCFA quantification by gas chromatography–mass spectrometry (GC-MS) [32,37,40] to assess alterations in microbial metabolism associated with cachexia. In the clinical studies, the gut microbiome was also analyzed using 16S rRNA gene sequencing, with additional targeted quantification of *Fn* by qPCR [42]. These analyses were performed on fecal samples collected pre-surgery in CRC patients, enabling the identification of microbial signatures associated with cachexia, including alterations in alpha diversity and relative abundances of specific genera [42,43].

All preclinical studies included male mice, with the most frequently used strains being BALB/c [34,35,36,37,39,40,41] and CD2F1 [29,30,31,32,33], while C57BL/6 mice were used in one study [38]. Mouse ages ranged from 5–8 weeks, except for one study that used 6-month-old mice [38]. Cachexia was predominantly induced through the C26 colon carcinoma model, involving subcutaneous injection of C26 tumor cells. Two studies utilized MC-38 colon cancer cells [33,38] to establish cachexia. The C26/CT26 model is widely recognized as a gold standard for investigating the pathogenesis and therapeutic interventions of cancer cachexia in CRC [44,45].

In the clinical studies, patient populations consisted of CRC patients in stages I–III, and both studies were derived from the same prospective cohort (ColoCare), but they differed in their specific research focus and sample sizes. One study included 87 participants [42], whereas the other included 103 participants [43]. Cachexia was assessed six months post-surgery using Fearon criteria, defined as >5% body weight loss over the past six months or a body mass index (BMI) < 20 kg/m^2^ with weight loss of >2% (Table 2).

### 3.3. Gut Microbiota Diversity in CRC Cachexia

Multiple studies using murine models of CRC cachexia reported significant alterations in gut microbiota diversity. Most studies found a significant decrease in alpha diversity metrics (such as Shannon, Chao, and Faith’s phylogenetic diversity indices) in cachectic mice compared to healthy controls, indicating reduced microbial richness and evenness [29,33,36,38,39,40,41]. Contrasting these findings, one study which also used the C26 model, reported a significant increase in alpha diversity [34]. Beta diversity analysis revealed distinct differences in microbial community composition between cachectic and control mice, confirming significant alteration in overall microbiota composition [29,31,36,37,38,41].

Regarding the diversity in the clinical studies, higher pre-surgical gut microbial alpha diversity was positively associated with cachexia onset at six months post-surgery, measured by the Shannon index (odds ratio = 2.00, 95% confidence interval = 1.07, 4.19), and for beta diversity, significant differences in overall microbiome composition were observed between cachectic and non-cachectic patients (Bray–Curtis distance, *p* = 0.01; unweighted UniFrac distance, *p* = 0.001) [43]. However, the second study did not discuss general microbial diversity metrics and focused exclusively on *Fn* abundance [42].

### 3.4. Taxonomic Shifts in Gut Microbiota in CRC Cachexia Models Compared to Healthy Controls

At the phylum level, cachectic mice frequently showed enrichment of Proteobacteria across studies [32,34]. Bacteroidetes were reported to be reduced in several studies, although some studies observed increases in specific genera within this phylum [33,34,36]. Findings for Firmicutes were inconsistent, with reports of both enrichment in three studies [34,35,36] and depletion in two studies [31,32]. Additionally, Deferribacteres and its representative genus *Mucispirillum* were also found to be increased in cachectic animals [33,34].

At the family level, Enterobacteriaceae showed a consistent expansion in cachectic mice in six studies [29,30,32,33,35,38]. Conversely, families with prominent SCFA-producing members, such as Lachnospiraceae and Ruminococcaceae, were mostly reduced [31,32,33,35,37], although one study reported increased Lachnospiraceae abundance [34]. Unclassified Borkfalkiaceae were reported to be decreased in one study [33], whereas Eubacteriaceae were increased in another study [35] (Table 3).

At the genus level, increased abundance of *Enterococcus* and *Bacteroides* was reported in cachectic mice in some studies [34,36]. Beneficial commensals were mostly depleted, including *Intestinimonas* [35], *Roseburia* [40], *Faecalibacterium*, *Butyricicoccus* [37], *Lachnoclostridium* [36], and *Prevotella* [34]. *Blautia* was increased in one study [35].

Cachexia was associated with an enrichment of several pathobionts, *Escherichia coli* [29], *Klebsiella oxytoca* [32], and *Parabacteroides goldsteinii/ASF 519* [29]. Conversely, *Lactobacillus johnsonii/gasseri* was reported to be reduced [29]. Notably, findings regarding Lactobacillaceae and its representative genus *Lactobacillus* were inconsistent. Several studies demonstrated increases [34,36], whereas others reported significant decreases [32,35,38,41]. *Xylanibacter rodentium* showed also a significant decrease in the cachectic group [33] (Table 4).

One clinical study reported that the abundance of *Fn* in pre-surgical fecal samples was significantly associated with cachexia onset at six months post-surgery; patients with high fecal *Fn* abundance exhibited a markedly increased risk of developing cachexia compared to those with negative or low *Fn* levels (OR = 4.82, 95% CI = 1.15–20.10, *p* = 0.03) [42]. In a separate investigation, the relative abundance of *Porphyromonas* was inversely associated with cachexia onset (OR = 0.51, 95% CI = 0.26–0.89, *p* = 0.03), with stronger inverse associations observed in males, patients receiving neoadjuvant treatment, those with rectal or stage III tumors, and patients with low dietary fiber intake. For *Actinomyces*, a suggestive inverse association was found with cachexia (OR = 0.72, 95% CI = 0.48–1.03, *p* = 0.08), particularly among patients younger than 65 years [43] (Table 5).

### 3.5. Quality Assessment

CAMARADES quality scores ranged from 5 to 6 out of 10 (Table S3), indicating moderate quality. All studies were peer-reviewed, used appropriate models, reported temperature control, obtained ethical approval, and disclosed conflicts of interest. Although randomization was reported in 10 of 13 studies, allocation concealment, blinded outcome assessment, and sample size calculations were consistently absent. These methodological limitations may have increased the risk of bias and may reduce confidence in the reproducibility of the preclinical findings. The risk of bias for the two clinical studies using Newcastle–Ottawa Scale indicated high-quality prospective cohorts (Table S4) [42,43]. However, the lack of baseline cachexia status assessment may have raised the possibility that the outcome was already present in some participants at the start of the defined follow-up [43].

## 4. Discussion

In this systematic review, we synthesized evidence on gut microbiome alterations in CRC-associated cachexia, with a primary focus on preclinical studies. Across mouse models, cachexia was frequently associated with reduced gut microbial diversity and shifts in microbial composition, including enrichment of Proteobacteria/Enterobacteriaceae and reductions in several SCFA-producing families such as Ruminococcaceae and Lachnospiraceae. However, the clinical evidence remains limited to two reports from the same cohort.

### 4.1. Mechanistic Insights into the Role of Dysbiosis in Cachexia Progression in Cachectic Mice

A central unresolved question is whether gut microbial dysbiosis in CRC-associated cachexia represents a downstream consequence of tumor burden or an active amplifier of disease progression.

Reduced food intake may partly contribute to dysbiosis in cachexia, since changes in diet have been shown to affect the composition of the gut microbiota [46]. However, pair-feeding experiments showed that major microbiota alterations occurred independently of food intake [29]. Furthermore, while mice pair-fed to cachectic mice (C26-PF) lost fat mass, cachectic mice lost muscle mass and developed a dysbiotic profile different from healthy pair-fed mice [30]. Consistently, in another study, *Klebsiella oxytoca* levels remained unchanged in pair-fed controls but were elevated in cachectic animals [31]. These findings suggest that anorexia alone does not account for the observed microbial changes and tumor-related factors and that pro-cachectic mediators, such as IL-6 and TNF-α, may also influence the intestinal environment and contribute to cachexia-associated dysbiosis.

Bindels et al. [30] suggested that tumor-driven IL-6 elevation may contribute to altered gut microbial dysbiosis in cachectic mice, particularly including Enterobacteriaceae expansion, which can promote pro-inflammatory signaling via LPS-TLR4 interactions [47]. Consistent with this, elevated serum lipopolysaccharide-binding protein (LBP) was observed in both cachectic mice and cancer patients. Notably, in other studies, this expansion was also correlated with a drop in cecal levels of butyrate and acetate [29,31] and was also found to be reduced with a synbiotic approach [29]. Additional mechanistic work suggested that Butyrate depletion was linked to altered host gut epithelial metabolism, characterized by reduced peroxisome proliferator-activated receptor gamma (PPAR-γ) signaling, which led to increased inducible nitric oxide synthase (iNOS) expression and host-derived nitrate production. Elevated nitrate was shown to favor the growth of Enterobacteriaceae such as *K. oxytoca*, with potential effects on gut barrier integrity and bacterial translocation [48]. Thus, Enterobacteriaceae enrichment may reflect and contribute to inflammatory changes in cachectic mice.

The recurrent findings of reduced of SCFA-producing bacteria support the microbial involvement in cachexia pathophysiology. Butyrate supplementation was found to counteract skeletal muscle atrophy by modulating the Akt/mTOR/Foxo3a and Fbox32/Trim63 pathways [49], and it preserved intestinal barrier function by upregulating tight junction proteins (ZO-1, occludin, claudin-1), while it reduced systemic inflammation and oxidative stress by suppressing macrophage infiltration and inhibiting pro-inflammatory cytokines (IL-6, TNF-α). The preclinical findings of reduced SCFA-producing bacteria in cachexia and the reduction of butyrate are supported by clinical data, where cachectic cancer patients showed lower faecal acetate compared to non-cachectic patients [37]. Notably, the loss of butyrate in clinical models was found to be associated with features of cachexia, including weight loss, elevated inflammatory factors, and metabolic dysregulation [36]. Furthermore, the observation that differential SCFAs negatively correlated with inflammatory mediators strengthens the argument that butyrate depletion exacerbates systemic inflammation in cachexia. This interpretation is consistent with the mechanism described by Ardis et al., linking gut dysbiosis, impaired barrier function, and inflammation, in which reduced SCFA availability may weaken epithelial barrier integrity, increase translocation of microbial products such as LPS, promote endotoxemia, and activate inflammatory signaling. This may increase systemic mediators such as IL-6 and TNF-α, which can activate muscle catabolic pathways, including NF-κB- and STAT3-related signaling, thereby contributing to skeletal muscle proteolysis [16]. Overall, these findings support a potential link between loss of SCFA-producing bacteria, altered microbial metabolites, and cachexia-related inflammation and muscle loss, but causality remains to be established.

**Figure 2:**
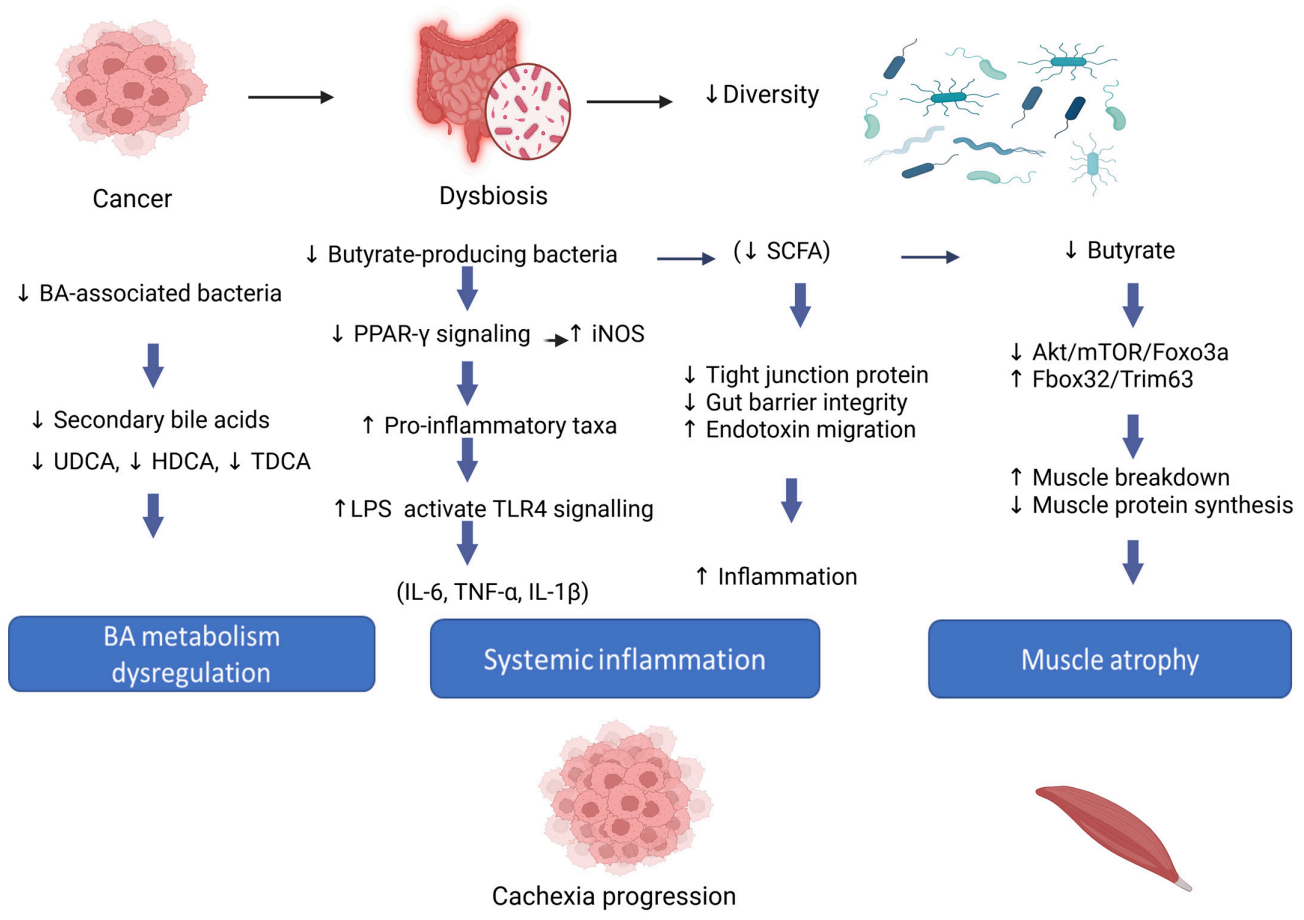
Putative association between gut microbiota and cachexia in CRC models: ↑ increased/upregulated; ↓ decreased/downregulated (figure created with BioRender.com).

Alterations in BA metabolism may represent one pathway linking gut microbial dysbiosis with metabolic changes in cancer cachexia. Kim et al. [41] found reduced abundance of microbial genes related to BA biosynthesis and Pötgens et al. [32] reported altered BA subsets and taurine accumulation, both indicative of disrupted microbial processing of BAs in cachectic mice. Moreover, Feng et al. [35] demonstrated BA metabolism dysregulation in both cachectic mice and human colon cancer patients, showing that decreased Lachnospiraceae and increased Enterobacteriaceae are associated with impaired microbial BA metabolism and activation of the FXR-FGF15 signaling pathway. More recently, Zou et al. [40] reported reduced levels of secondary BAs, including ursodeoxycholic acid (UDCA) and hyodeoxycholic acid (HDCA), in cecal contents of cachectic colon cancer mice, together with depletion of BA-associated genera such as *Alistipes*, *Eubacterium*, and *Roseburia*. Thibaut et al. [33] also reported early depletion of taurodeoxycholic acid (TDCA) and diminished microbial 7α-dehydroxylation activity prior to the onset of overt cachectic symptoms, suggesting that microbial and metabolic perturbations occur before cachexia develops and may represent initiating events rather than downstream consequences. Although the specific BA species and bacterial taxa differed across studies, these findings collectively suggest that impaired microbial secondary BA metabolism may be associated with CRC cachexia (Figure 2).

Regarding the role of *Lactobacillus* in CRC associated cachexia, decreased levels of the Lactobacillaceae family and the *Lactobacillus* genus in C26 tumor-bearing mice were observed, while synbiotic supplementation with *Lactobacillus reuteri 100-23* restored microbial balance, reduced muscle wasting, improved intestinal barrier integrity, and decreased morbidity [29]. Similarly, Kim et al. [41] found reduced *Lactobacillus* abundance in CT26-induced cachectic mice and noted that supplementation with *Lactobacillus* strains attenuated systemic inflammation and muscle atrophy.

This finding was further supported by Qiu et al. [39], who found that engineered *Lactobacillus* strains significantly improved the quality of life in cachectic mice by restoring gut microbial balance and increased the production of SCFAs. Two other studies also reported that enhancing *Lactobacillus* levels in colon cancer models counteracted muscle atrophy and lowered systemic inflammation, highlighting the therapeutic potential of these bacteria in mitigating cachexia hallmarks [30,31]. Overall, these findings suggest that selected *Lactobacillus*-based interventions may modulate cachexia-related phenotypes in preclinical models.

Several studies found an increase in Proteobacteria in the cachectic group, including flagellated microbes. Flagellin is recognized as a ligand for toll-like receptor 5 (TLR5); upon binding, it activates intracellular signaling pathways that lead to the transcription of pro-inflammatory cytokines, including IL-6. Pekkala et al. [34] explored this mechanism by exposing C26 cancer cells to flagellin; the resulting conditioned medium, enriched in inflammatory mediators such as IL-6 and C-C motif chemokine ligand 2 (CCL2), induced deterioration and reduced myotube number in murine C2C12 myotubes. This finding may suggest a possible link between flagellin-associated inflammatory signaling and muscle deterioration in preclinical cachexia models.

### 4.2. Reconciling Contradictions

The overall microbial diversity of the gut microbiota was generally found to be decreased in cachectic mice, reflecting a state of profound gut dysbiosis [50]. In contrast, in the included clinical study, higher pre-surgical gut microbial alpha diversity was positively associated with cachexia onset at six months post-surgery. This observation contrasts with prior studies in other cancer types, such as pancreatic and gastric cancer, where cachexia was more commonly associated with lower alpha diversity or no significant difference [9,51]. Nonetheless, increased diversity does not necessarily indicate a healthier microbiome but may instead suggest a more complex dysbiosis that promotes cachexia or may reflect higher inter-individual variability, rather than restored eubiosis. One other possible explanation for increased alpha diversity, proposed by Ilozumba et al., is the influence of baseline adiposity, as the non-cachectic group had a higher proportion of patients with obesity, a condition that has often been associated with lower gut microbial diversity [43]. In CRC, tumor-related changes, inflammation, and altered diet can disrupt the normal gut ecosystem, leading to an increase in both beneficial and potentially pathogenic or pro-inflammatory taxa. This results in a more diverse but functionally imbalanced microbiome [52]. Despite conflicting alpha-diversity findings, beta-diversity analyses generally showed separation between cachectic and non-cachectic groups. This suggests that cachexia is associated with differences in overall gut microbial community structure, even when within-sample diversity does not change consistently.

At the phylum level, findings for Firmicutes were contradictory, with some studies reporting increased abundance and others reporting decreased abundance. This inconsistency is not unexpected, as Firmicutes is a broad and functionally diverse phylum, which includes potentially beneficial SCFA-producing bacteria as well as taxa with different functional roles. While several included studies found decreased *Lactobacillus* in cachectic mice, Liu et al. [36] reported an increase in *Lactobacillus* during CRC cachexia and observed positive correlations between *Lactobacillus* levels and elevated pro-inflammatory cytokines (IL-1β, IL-6, TNF-α) and LPS levels, suggesting that this may serve as a biomarker of the cachectic state. Similarly, Pekkala et al. [34] reported increases in the Lactobacillaceae family and the *Lactobacillus* genus, speculating that this may represent a compensatory response to reduced energy intake, or that tumor-associated biofilms could favor *Lactobacillus* colonization. These inconsistent findings may arise from differences in host background, nutritional status, and strain-specific functional capacities. In addition, taxa such as *Lactobacillus* include many different species with distinct functional properties. Therefore, changes observed at the family or genus level may not always reflect the same biological role across studies. Thus, understanding these nuances is essential.

Regarding the secondary BA metabolism alteration, while Feng et al. [35] highlighted the contribution of bile salt hydrolase (BSH) deficiency driven by *Lachnospiraceae*, Thibaut et al. [33] used stable isotopes to show that BSH activity was actually unchanged and that the true deficit lay in the subsequent 7α-dehydroxylation step, probably driven by the loss of different bacteria such as *Xylanibacter rodentium* and unclassified *Borkfalkiaceae*. Despite these differences, the functional outcome is consistent; reduced microbial capacity to produce secondary BAs, leading to BAs dysregulation and hepatic metabolic stress, highlights BA-targeted therapies as promising strategies to restore metabolic balance [40].

### 4.3. Clinical Evidence

The studies by Ilozumba et al. [42,43] were the first to investigate associations between the gut microbiome and the onset of cachexia in CRC patients. The first study identified a strong positive association between high pre-surgical fecal *Fn* abundance and the increased risk of cachexia onset at six months post-surgery. *Fn*, a putatively oncogenic bacterium, stimulates the expression of genes encoding key pro-inflammatory cytokines [53]. However, it also produces SCFAs, typically anti-inflammatory, complicating the interpretation of its functional role [54]. While *Fn* enrichment was linked to cachexia onset, this bacterium has also been found elevated in CRC independent of cachexia [55]. Therefore, it remains difficult to separate cachexia-specific microbiota changes from tumor-driven alterations.

The second study found that lower relative abundances of *Porphyromonas* and *Actinomyces* were associated with cachexia onset. This finding is difficult to interpret because the associations were observed at the genus level, and different species within these genera may have distinct functional roles. For example, species like *Porphyromonas gingivalis* are typically linked to pro-inflammatory roles and decreased survival in CRC. However, the observed inverse association suggests that other *Porphyromonas* species might modulate the immune response and reduce systemic inflammation, thereby potentially counteracting muscle wasting [56,57].

In comparison with other cancer types, Ni et al. reported significant beta-diversity differences in lung cancer cachexia, suggesting that overall gut microbial community structure differed between cachectic and non-cachectic patients. This is consistent with the CRC clinical studies, in which beta-diversity differences were also observed. However, the specific microbial signatures differed between cancer types. In lung cancer cachexia, Ni et al. reported depletion of *Prevotella copri* and *Lactobacillus gasseri* and enrichment of *Klebsiella oxytoca* and *Methanobrevibacter smithii*. These findings suggest that gut microbiome alterations may be relevant across cancer cachexia, but that the specific taxa involved may depend on cancer type [58].

Since these two included studies originated from the same clinical cohort, the associations involving *Porphyromonas*, *Actinomyces*, and *Fn* may reflect cohort-specific effects. Therefore, they should be considered exploratory rather than conclusive and require validation in independent CRC cohorts with higher-resolution microbiome profiling.

### 4.4. Limitations

Human and animal models differ in gastrointestinal anatomy, microbial ecology, and systemic physiology. Mice possess a proportionally larger cecum, shorter colon and distinct bile acid composition, posing significant challenges for translating preclinical findings to humans, and cachectic patients often present with more advanced disease or receive treatments that themselves alter microbial composition,. Most of the included studies used ectopic C26 models, in which tumor cells were implanted subcutaneously rather than developing within the colorectal mucosa. Therefore, these models may not have fully reproduced the local tumor–microbiota interactions. Additionally, a major limitation is the restricted scope of the available clinical evidence and the lack of external validation. Only two clinical studies were identified, and both were derived from the same ColoCare cohort, which included CRC patients from the United States and Germany.

Moreover, the cachexia assessments at six months post-surgery may have been confounded by post-operative inflammation or adjuvant treatments. Although the included clinical studies attempted to account for some potential confounding factors, for example, by excluding patients with recent antibiotic use and adjusting for dietary factors such as energy and fiber intake, residual confounding remains possible due to missing data. Therefore, the clinical findings may not be generalizable to other patient populations and should be treated as exploratory and hypothesis-generating until validated in independent clinical cohorts.

Finally, the use of 16S rRNA gene sequencing restricts taxonomic resolution to the genus level and limits functional characterization of the microbiome. Therefore, future studies should incorporate higher-resolution approaches, including deep amplicon sequencing, shotgun metagenomic sequencing, metatranscriptomics, metabolomics, and integrated multi-omics analyses, to better characterize microbial functions, strain-level variation, and microbiome-derived metabolites involved in CRC-associated cachexia.

## 5. Conclusions

While tumor burden is likely to trigger the initial inflammatory insult, the resulting dysbiosis may further amplify inflammatory signaling and metabolic dysfunction associated with cachexia, although its causal role remains unclear. Microbiota-directed interventions have shown promising effects in reducing cachexia-related features in experimental models; however, they are not yet clinically established for CRC-associated cachexia in humans. Future research should prioritize well-powered longitudinal human cohorts, independent validation, and higher-resolution multi-omics approaches, in order to strengthen confidence in these associations and clarify their directionality and clinical relevance.

## Figures and Tables

**Table 1:** Characteristics of preclinical studies included in the systematic review.

Author (Year)	Country	Study Design	Mouse Model (Age)/Tumor Cell Line/Tumor Model (Ectopic vs. Orthotopic)	Sample Size	Cachexia Definition	Microbiota Analysis Method
Bindels (2016) [29]	Belgium, United Kingdom, France, and Canada	Experimental study	Male CD2F1 (8 weeks old) Colon carcinoma 26 (C26) cells Ectopic	6 vs. 8 mice	Loss of fat mass and muscle atrophy	16S rRNA
Bindels (2018) [30]	Belgium and France	Experimental study	Male CD2F1 (7 weeks old) Colon carcinoma 26 (C26) cells Ectopic	7 vs. 8 mice	Decreased food intake and loss of body weight, due to muscle atrophy and later, to adipose tissue loss.	16S rRNA qPCR
Pötgens (2018) [31]	Belgium	Experimental study	Male CD2F1 (7 weeks old) Colon carcinoma 26 (C26) cells Ectopic	7 vs. 8 mice	Body weight loss, weakness, muscle atrophy, fat depletion and decreased food intake	16S rRNA qPCR
Pekkala (2019) [34]	Finland	Experimental study	Male BALB/cAnNCrl mice (5–6-week-old) Colon 26 carcinoma (C26) cell line Ectopic	9 vs. 7 mice	Body weight decreased, muscle atrophy, and fat depletion	16S rRNA
Feng (2021) [35]	China	Experimental study	Male BALB/c mice (6–8 weeks old) C26 colon tumor cells Ectopic	8 mice	>5% body weight loss, reduced muscle mass, fat depletion, elevated inflammatory markers (LPS, IL-6, TNF-α, IL-1β)	16S rRNA
Pötgens (2021) [32]	Belgium & United Kingdom	Experimental study	Male CD2F1 mice (6–7 weeks old) Colon carcinoma 26 (C26) cells Ectopic	8 mice	The cachexia mouse model exhibited weight loss, muscle atrophy, and elevated inflammatory factors	16S rRNA Metabolomics analysis
Kim (2023) [41]	Korea	Experimental study	Male BALB/c mice (6 weeks old) C26 colon tumor cells Ectopic	10–13 mice	Loss of adipose and muscle tissues and decreased carcass weight (after tumor removal)	16S rRNA
Liu (2023) [36] a	China	Experimental study (in vivo & in vitro)	Male BALB/c mice (6–8 weeks old) C26 colon tumor cells Ectopic	10 mice	Progressive weight loss, muscle atrophy, decreased grip strength and elevated inflammatory markers (LPS, IL-6, TNF-α, IL-1β).	16S rRNA
Liu (2023) [37] b	China	Experimental study	Male BALB/c mice (6–8 weeks) C26 colon tumor cells Ectopic	6 mice	Skeletal muscle atrophy and weight loss	16S rRNA
Thibaut (2025) [33]	Belgium and United Kingdom	Experimental study	Male CD2F1 mice (7 weeks)/C26 colon tumor cells/ectopic; Male C57BL/6 mice (8 weeks)/murine MC38 colorectal cancer cells/ectopic; Male NOD scid gamma (NSG) mice (8 weeks)/human HCT116 colorectal cancer cells/ectopic	8 mice	Body weight and fat mass loss, muscle atrophy	16S rRNA
Qiu (2025) [39]	China	Experimental study	BALB/c mice CT26 tumor cell line Ectopic	6 mice	Reduction in grip strength, loss of muscle mass	16S rRNA
Jin (2025) [38]	China	Experimental study	C57BL/6 mice (6-month-old) MC-38 colon cancer cells Orthotopic	6 mice	Weight loss, muscle mass reduction	16S rRNA
Zou (2026) [40]	China	Experimental study	Male BALB/c mice, (5 weeks old) CT26 tumor cell line Ectopic	10–12 mice	Reduced food intake, significant involuntary weight loss, loss of skeletal muscle and fat mass	shotgun metagenomics, metabolomic analysis

**Table 2:** Characteristics of clinical studies included in the systematic review.

Author (Year)	Country	Study Design	Population	Cancer Stage	Cachexia Assessment	Microbiota Analysis Method
Ilozumba (2024) [42]	USA and Germany	Prospective cohort (ColoCare)	Total: 87 patients Cachectic: 39 (45%) Non-cachectic: 48 (55%)	Stages I–III CRC	Fearon criteria	16S rRNA gene sequencing *Fusobacterium nucleatum* via qPCR
Ilozumba (2025) [43]	USA and Germany	Prospective cohort (ColoCare)	Total: 103 patients Cachectic: 44 (43%) Non-cachectic: 59 (57%)	Stages I–III CRC	Fearon criteria	16S rRNA gene sequencing

**Table 3:** Gut microbiota changes in the cachectic mice compared to the healthy mice by phylum and family.

Taxonomic Level Taxa	Study	Direction of Change	Quantitative Measures (Cachectic vs. Control)
**Phylum**			
Firmicutes	Pötgens et al. [31]	↓ Decrease	NR
	Pekkala et al. [34]	↑ Increase	*p* = 0.046
	Pötgens et al. [32]	↓ Decrease	q < 0.05
	Feng et al. [35]	↑ Increase	NR
	Liu et al. [36]	↑ Increase	NR
	Thibaut et al. [33]	↑ Increase	q < 0.05
Proteobacteria	Pekkala et al. [34]	↑ Increase	*p* = 0.046
	Pötgens et al. [32]	↑ Increase	q < 0.05
	Thibaut et al. [33]	↑ Increase	q < 0.05
	Jin [38]	↑ Increase	NR
Bacteroidetes	Pekkala et al. [34]	↓ Decrease	*p* = 0.046
	Liu [36]	↓ Decrease	NR
	Thibaut et al. [33]	↓ Decrease	q = 0.002
Deferribacteres	Pekkala et al. [34]	↑ Increase	*p* = 0.046
	Thibaut et al. [33]	↑ Increase	q = 0.02
**Family**			
Enterobacteriaceae	Bindels et al. [29]	↑ Increase	*p* < 0.001
	Bindels et al. [30]	↑ Increase	*p* < 0.05
	Pötgens et al. [32]	↑ Increase	q < 0.05
	Feng et al. [35]	↑ Increase	NR
	Thibaut et al. [33]	↑ Increase	NR
	Jin et al. [38]	↑ Increase	NR
Lachnospiraceae	Pötgens et al. [31]	↓ Decrease	*p* = 0.009
	Pekkala et al. [34]	↑ Increase	*p* < 0.001
	Feng et al. [35]	↓ Decrease	*p* < 0.05
	Liu et al. [37]	↓ Decrease	*p* < 0.05
	Thibautet al. [33]	↓ Decrease	NR
Ruminococcaceae	Pötgens et al. [31]	↓ Decrease	*p* < 0.05
	Pötgens et al. [32]	↓ Decrease	NR
	Liu et al. [37]	↓ Decrease	*p* < 0.05
	Thibaut et al. [33]	↓ Decrease	NR
Lactobacillaceae	Pekkala et al. [34]	↑ Increase	q < 0.05
	Pötgens et al. [32]	↓ Decrease	q < 0.05
Borkfalkiaceae (unclassified)	Thibaut et al. [33]	↓ Decrease	*p* = 0.005
Clostridiales incertae sedis XIII	Thibaut et al. [33]	↑ Increase	q < 0.01
Deferribacteraceae	Thibaut et al. [33]	↑ Increase	q < 0.05
Eubacteriaceae	Feng et al. [35]	↑ Increase	*p* < 0.05
Streptococcaceae	Thibaut et al. [33]	↑ Increase	q < 0.01

**Note:** ↑ indicates increased cachexia group ↓ indicates decreased cachexia group; NR, not reported.

**Table 4:** Gut microbiota changes in the cachectic mice compared to the healthy mice by genus, species, and strain.

Taxonomic Level Taxa	Study	Direction of Change	Quantitative Measures (Cachectic vs. Control)
**Genus**			
*Lactobacillus*	Pekkala et al. [34]	↑ Increase	*p* = 0.008
	Feng et al. [35]	↓ Decrease	NR
	Liu et al. [36]	↑ Increase	*p* < 0.05
	Kim et al. [41]	↓ Decrease	NR
	Qiu et al. [39]	↓ Decrease	*p* = 0.031
	Jin et al. [38]	↓ Decrease	NR
*Bacteroides*	Pekkala et al. [34]	↑ Increase	*p* = 0.030
	Zou et al. [40]	↑ Increase	*p* < 0.05
	Liu et al. [36]	↑ Increase	*p* < 0.05
*Enterococcus*	Pekkala et al. [34]	↑ Increase	*p* = 0.011
	Liu et al. [36]	↑ Increase	*p* < 0.05
*Eubacterium*	Kim et al. [41]	↓ Decrease	NR
	Zou et al. [40]	↓ Decrease	*p* < 0.05
	Liu et al. [37]	↓ Decrease	*p* < 0.05
*Intestinimonas*	Feng et al. [35]	↓ Decrease	NR
	Kim et al. [41]	↓ Decrease	NR
*Mucispirillum*	Pekkala et al. [34]	↑ Increase	*p* = 0.037
	Thibaut et al. [33]	↑ Increase	q < 0.05
*Prevotella*	Pekkala et al. [34]	↓ Decrease	*p* = 0.011
	Thibaut et al. [33]	↓ Decrease	q < 0.05
*Blautia*	Feng et al. [35]	↑ Increase	NR
*Alistipes*	Zou et al. [40]	↓ Decrease	*p* < 0.05
*Clostridia vadin* *BB60 gp*	Feng et al. [35]	↓ Decrease	NR
*Faecalibacterium*	Liu et al. [37]	↓ Decrease	*p* < 0.05
*Lachnoclostridium*	Liu et al. [36]	↓ Decrease	*p* < 0.05
*Muribaculum*	Liu et al. [36]	↓ Decrease	*p* < 0.05
*Parabacteroides*	Thibaut et al. [33]	↑ Increase	q < 0.05
*Roseburia*	Liu et al. [37]	↓ Decrease	*p* < 0.05
	Zou et al. [40]	↓ Decrease	*p* < 0.05
*Sporobacter* spp.	Thibaut et al. [33]	↓ Decrease	q < 0.05
*Streptococcus*	Thibaut et al. [33]	↑ Increase	q < 0.05
*Turicibacter*	Feng et al. [35]	↓ Decrease	NR
**Species**			
*Escherichia coli*	Bindels et al. [29]	↑ Increase	NR
*Klebsiella oxytoca*	Pötgens et al. [31]	↑ Increase	*p* < 0.05
*Lactobacillus johnsonii/gasseri*	Bindels et al. [29]	↓ Decrease	*p* = 0.06
*Parabacteroides goldsteinii*	Jin et al. [38]	↑ Increase	NR
*Xylanibacter rodentium*	Thibaut et al. [33]	↓ Decrease	q < 0.05
**Strain**			
*Parabacteroides goldsteinii/ASF 519*	Bindels et al. [29]	↑ Increase	*p* < 0.001
**Uncultured**			
*Lachnospiraceae* *UCG-004*	Kim et al. [41]	↓ Decrease	NR

**Note:** ↑ indicates increased cachexia group ↓ indicates decreased cachexia group; NR, not reported.

**Table 5:** Associations Between Pre-Surgery Gut Microbiota and Cachexia Onset at 6 Months Post-Surgery in CRC Patients (ColoCare Study).

Taxonomic Level Taxa	Study	OR (95%CI)	Cachexia Risk	Notes
**Species**				
*Fusobacterium nucleatum*	Ilozumba et al. [42]	4.82 (1.15–20.10)	↑ Increase	A priori CRC-associated
**Genus**				
*Porphyromonas*	Ilozumba et al. [43]	0.51 (0.26–0.89)	↓ Decrease	Protective
*Actinomyces*	Ilozumba et al. [43]	0.72 (0.48–1.03)	↓ Decrease	Cachexia-relevant

**Note:** ↑ indicates increased cachexia group ↓ indicates decreased cachexia group.

## Data Availability

No new data were generated in this study. All data analyzed in this systematic review were derived from previously published studies.

## Supplementary Materials

The following supporting information can be downloaded at: https://www.mdpi.com/article/10.3390/cancers18152387/s1, Tables S1: Search terms used in this systematic literature review; Table S2: Studies Appearing to Meet the Inclusion Criteria but Were Excluded; Table S3: Methodological Quality Assessment of Included Preclinical Studies Using the CAMARADES checklist; Table S4: Risk of bias based on the NOS for cohort studies.
