# Supplementary material for: The Association Between Gut Microbiome and Cachexia in Colorectal Cancer: A Systematic Review
Source: Cancers (Basel). 2026 Jul 24;18(15):2387. doi: 10.3390/cancers18152387 (PMC13463872; doi:10.3390/cancers18152387)
Supplement: Supplementary file 1 [file cancers-18-02387-s001.zip › cancers-4429033-supplementary.pdf]

Supplementary Table S1. Search terms used in this systematic literature review.

Database	Search string
PubMed	(("Neoplasms"[Mesh] OR cancer*[tiab] OR tumor*[tiab] OR carcinoma*[tiab] OR neoplasm*[tiab] OR "colorectal cancer"[tiab] OR CRC[tiab])<br>AND<br>("Cachexia"[Mesh] OR cachexia[tiab] OR "cancer cachexia"[tiab] OR "muscle wasting"[tiab] OR "muscle atrophy"[tiab] OR "weight loss"[tiab] OR sarcopenia[tiab])<br>AND<br>("Gastrointestinal Microbiome"[Mesh] OR "Microbiota"[Mesh] OR "gut microbiota"[tiab] OR "intestinal microbiota"[tiab] OR microbiome[tiab] OR dysbiosis[tiab] OR microflora[tiab] OR "gut bacteria"[tiab])
Scopus	(TITLE-ABS-KEY(cancer OR tumor* OR carcinoma* OR neoplasm*)<br>AND<br>TITLE-ABS-KEY(cachexia OR "cancer cachexia" OR "muscle wasting" OR "weight loss" OR "muscle atrophy" OR sarcopenia)<br>AND<br>TITLE-ABS-KEY("gut microbiota" OR "intestinal microbiota" OR microbiome OR dysbiosis OR microflora OR "gut bacteria" OR "gut microbial composition"))
Web of Science	TS=((cancer OR tumor* OR carcinoma* OR neoplasm*)<br>AND<br>(cachexia OR "cancer cachexia" OR "muscle wasting" OR "weight loss" OR "muscle atrophy" OR sarcopenia)<br>AND<br>("gut microbiota" OR "intestinal microbiota" OR microbiome OR dysbiosis OR microflora OR "gut bacteria" OR "gut microbial composition"))

Supplementary Table S2. Studies Appearing to Meet the Inclusion Criteria but Were Excluded.

Reason	Study
No microbial comparative analysis (n=2)	1. Costa, R.G.F., et al., *Cancer cachexia induces morphological and inflammatory changes in the intestinal mucosa.* J Cachexia Sarcopenia Muscle, 2019. **10**(5): p. 1116-1127. [22]<br><br>2. Jiang, Y., et al., *Bacterial translocation contributes to cachexia and its possible pathway in patients with colon cancer.* J Clin Gastroenterol, 2014. **48**(2): p. 131-7. [23]
Comparator group is treatment (n=5)	1. Alsholi, D.M., et al., *Lactobacillus rhamnosus Attenuates Cisplatin-Induced Intestinal Mucositis in Mice via Modulating the Gut Microbiota and Improving Intestinal Inflammation.* Pathogens, 2023. **12**(11). [24]<br><br>2. Huang, G., et al., *Ginsenosides Rb3 and Rd reduce polyps formation while reinstate the dysbiotic gut microbiota and the intestinal microenvironment in Apc(Min/+) mice.* Sci Rep, 2017. **7**(1): p. 12552. [25]<br><br>3. Wang, J.L., et al., *Resistant Starch-Encapsulated Probiotics Attenuate Colorectal Cancer Cachexia and 5-Fluorouracil-Induced Microbial Dysbiosis.* Biomedicines, 2024. **12**(7). [26]<br><br>4. Sakakida, T., et al., *Water-soluble dietary fiber alleviates cancer-induced muscle wasting through changes in gut microenvironment in mice.* Cancer Sci, 2022. **113**(5): p. 1789-1800. [27]<br><br>5. Varian, B.J., et al., *Beneficial bacteria inhibit cachexia.* Oncotarget, 2016. **7**(11): p. 11803-16. [28]

Supplementary Table S3. Methodological Quality Assessment of Included Preclinical Studies Using the CAMARADES checklist.

CAMARADES CHECKLIST	Bindels (2016)	Bindels (2018)	Pötgens (2018)	Pekkala (2019)	Feng (2021)	Pötgens (2021)	Kim (2023)	Liu (2023)	Liu (2023)	Thibaut (2025)	Qiu (2025)	Jin (2025)	Zou (2026)
Peer-reviewed journal	Y	Y	Y	Y	Y	Y	Y	Y	Y	Y	Y	Y	Y
Statement of control of temperature	Y	Y	Y	Y	Y	Y	Y	Y	Y	Y	Y	Y	Y
Randomization of treatment or control	N	Y	Y	Y	Y	Y	Y	Y	Y	Y	N	N	Y
Allocation concealment	N	N	N	N	N	N	N	N	N	N	N	N	N
Blinded assessment of outcome	N	N	N	N	N	N	N	N	N	N	N	N	N
Avoidance of anesthetics with marked intrinsic properties	N	N	N	N	N	N	N	N	N	N	N	N	N
Model Selection	Y	Y	Y	Y	Y	Y	Y	Y	Y	Y	Y	Y	Y
Sample size calculation	N	N	N	N	N	N	N	N	N	N	N	N	N
Statement of compliance with regulatory requirements	Y	Y	Y	Y	Y	Y	Y	Y	Y	Y	Y	Y	Y
Statement regarding possible conflict of interest	Y	Y	Y	Y	Y	Y	Y	Y	Y	Y	Y	Y	Y
**Total (out of10)**	**5**	**6**	**6**	**6**	**6**	**6**	**6**	**6**	**6**	**5**	**5**	**5**	**6**

Note: Y, yes; N, no. CAMARADES, Collaborative Approach to Meta-Analysis and Review of Animal Data from Experimental Studies.

Supplementary Table S4. Risk of bias based on the NOS for cohort studies.

	Selection (max. 4)				Comparability (max. 2)	Outcome (max. 3)			
**Study**	Represen-tativeness	Selection of the non-exposed group	Ascertain-ment of exposure	Outcome not present at start of study	Comparability of cohorts on the basis of design / analysis	Assessment of outcome	Follow-up long enough	Follow-up adequacy	**Overall quality**
**Ilozumba (2024)**	★	★	★	★	★★	★	★	★	High (9)
**Ilozumba (2025)**	★	★	★	–	★★	★	★	★	High (8)

Note: NOS, Newcastle–Ottawa Scale. High quality, 7–9 stars; moderate quality, 4–6 stars; low quality, 0–3 stars.
